# Ion annotation-assisted analysis of LC-MS based metabolomic experiment

**DOI:** 10.1186/1477-5956-10-S1-S8

**Published:** 2012-06-21

**Authors:** Rency S Varghese, Bin Zhou, Mohammad R Nezami Ranjbar, Yi Zhao, Habtom W Ressom

**Affiliations:** 1 Department of Oncology, Georgetown University, DC, USA; 2 Department of Electrical and Computer Engineering, Virginia Tech, Falls Church, VA, USA; 3 Department of Biostatistics, Bioinformatics, and Biomathematics, Georgetown University, DC, USA

## Abstract

**Background:**

Analysis of multiple LC-MS based metabolomic studies is carried out to determine overlaps and differences among various experiments. For example, in large metabolic biomarker discovery studies involving hundreds of samples, it may be necessary to conduct multiple experiments, each involving a subset of the samples due to technical limitations. The ions selected from each experiment are analyzed to determine overlapping ions. One of the challenges in comparing the ion lists is the presence of a large number of derivative ions such as isotopes, adducts, and fragments. These derivative ions and the retention time drifts need to be taken into account during comparison.

**Results:**

We implemented an ion annotation-assisted method to determine overlapping ions in the presence of derivative ions. Following this, each ion is represented by the monoisotopic mass of its cluster. This mass is then used to determine overlaps among the ions selected across multiple experiments.

**Conclusion:**

The resulting ion list provides better coverage and more accurate identification of metabolites compared to the traditional method in which overlapping ions are selected on the basis of individual ion mass.

## Background

### Introduction

Metabolomics is a field of omics science concerned with the comprehensive characterization of small molecule metabolites found in cells, tissues, biofluids, and organisms. Because metabolomics deals with small molecule products of gene, protein, and environmental interactions, it provides complementary information to what is normally obtained via genomics, transcriptomics, and proteomics. As a consequence, metabolomics is playing an increasingly important role in systems biology.

Based on prevalent practices, there are two approaches to conduct a metabolomic experiment (targeted and untargeted). In the targeted approach, compounds are first identified prior to quantification for difference detection. In untargeted metabolomics, spectral features from two or multiple sets of samples are processed chemometrically to select significant differences. The compounds contributing to these differences are then identified. Thus, in contrast to the targeted approach, untargeted metabolomics aims to detect as many metabolites as possible to maximize the opportunity of identifying compounds that are dysregulated in a particular biological condition.

Mass spectrometry (MS) is a favorable technology for metabolomic study because of its improved accuracy, sensitivity, and coverage. Chromatography is often coupled to mass spectrometer to achieve further separation of the sample. Both gas chromatography (GC) and liquid chromatography (LC) have been used in metabolomics studies [1,2]. Liquid chromatography coupled to mass spectrometry (LC-MS) has gained increasing application in untargeted metabolomic studies partly because it allows separation of compounds without derivatization. Electrospray ionization (ESI) is commonly used in LC-MS to form intact molecular ions and facilitate the initial identification of metabolites.

In large metabolic biomarker discovery studies involving hundreds of samples, it is necessary to conduct multiple experiments, each involving a subset of the samples, to avoid extremely long analytical runs or preparation of a large number of samples at once. The ions selected from each experiment are then compared to determine overlapping ions. One of the challenges in comparing the ion lists is the lack of recognition of a large number of derivative ions such as isotopes, adducts, and fragments. For example, a mass-based search could lead to wrong metabolite identification, if these derivatives are not recognized. This is due to the assumption by the databases that each one of the derivatives is a distinct molecular ion. Recognition of ions originating from the same metabolite improves the accuracy of metabolite identification. Also, it will facilitate the comparison of overlapping ions from multiple metabolomic experiments by comparing their monoisotopic mass instead of their individual masses.

In this paper, we analyze LC-MS data from multiple metabolomic experiments in positive and negative modes. Ions that share the same monoisotopic mass are grouped on the basis of their annotation information. The monoisotopic mass is then used to compare the ions across different datasets. The resulting ion list provides better coverage and more accurate identification of metabolites and thereby helps in the acceleration of the downstream bioanalysis. We compare the results obtained from our proposed method against the traditional method of combining the peaks based on their ion mass prior to identification. In the following, we explain the steps involved in the analysis of LC-MS data in a typical untargeted metabolomic study.

### LC-MS data preprocessing

Data preprocessing transforms raw data files into representation that facilitates easy access to characteristics of each observed ion including mass-to-charge ratio (*m*/*z*), retention time of the ion, and ion intensity measurement. Peak detection converts the raw data to an ion list. The ions from different samples are then matched and their retention time aligned to enable the comparison of multiple samples. Normalization corrects for any systematic bias across samples, which may be induced during the sample preparation and data acquisition. In addition, depending on the condition of the data, outlier screening, filtering and baseline correction are performed before peak detection to exclude LC-MS datasets which differ substantially from others, to enhance the signal-to-noise ratio, and correct for baseline shift. Several tools for LC-MS data preprocessing have been developed in the past years, such as MarkerLynx, MetAlign [3], XCMS [4], MetaboAnalyst [5], and MZmine [6,7]. Other packages, some of them specific for LC-MS-based metabolomics, have been reviewed in ref. [8].

LC-MS based metabolomic experiments yield large numbers of peaks. However, only a small fraction of which can be identified by database matching. Also, many of the molecules detected by mass-based approaches could be wrong if isotopes, fragments, and adducts are not recognized and are treated as monoisotipic ions formed during the ionization procedure [9]. Since each metabolite can give rise to multiple ions corresponding to derivative molecules, LC-MS runs often contain a large number of ions. Thus, only a fraction is of interest as others are derivatives of the same set of metabolites. Deisotoping or clustering the isotopic ions that correspond to the same compound is necessary prior to mass-based metabolite identification. Treating each observed ion as a unique metabolite could lead to wrong metabolite identification.

Different combinations of peak filtering and deisotoping approaches have recently been made available as part of various software packages, including XCMS [4,10]. SIRIUS [11] uses the isotope distribution and mass information to obtain the sum formula, and provides methods for isotope pattern simulation. Decon2LS [12] works on the raw data instead of the peak list. It is based on an algorithm called thorough high resolution analysis of spectra by Horn (THRASH) [13] that contains modules for background correction, determination of charge states, calculation of theoretical profiles and for subsequent fitting of observed isotopic results. Decon2LS analyzes mass spectral data for each scan, and deletes isotopic peaks, leaving a list of monoisotopic peaks for subsequent analysis. MZmine 2 has a peak list deisotoping algorithm that works with compounds which have few isotopes with continuously decreasing intensity.

### Difference detection

Following data preprocessing, statistical and machine learning methods are typically used to identify significant differences in metabolic changes between distinct biological groups. To find potential biomarkers, ion intensities are compared between distinct groups of samples such as healthy individuals vs. patients or cases vs. controls. Difference detection allows the identification of features that may otherwise be obscured by biological variability not related to disease.

Statistical methods for difference detection include parametric methods such as t-test and analysis of variance (ANOVA) and non-parametric methods such as the Wilcoxon rank-sum test. Because thousands of ions can be simultaneously profiled in an untargeted metabolomic studies, the multiple hypothesis testing problem will result in a high chance of false discovery even with a small p-value threshold. The selection of a reasonable FDR threshold controls the proportion of false positives among all features called significant. This is usually appropriate because one wants to find as many truly different features as possible with relatively few false positives. A q-value for each peak can be evaluated which is the minimum acceptable FDR at which that peak is called significant [14]. The Wilcoxon rank-sum test ranks each peak using an absolute value of the u-statistic of a two-sample unpaired Wilcoxon test, commonly known as Mann-Whitney. In statistics, the Mann-Whitney U test (also called the Mann Whitney Wilcoxon test) is a non-parametric statistical hypothesis test for assessing whether two independent samples of observations have equally large values.

### Metabolite identification and verification

One of the major challenges in metabolomic studies is the identification of metabolites. Compared to peptide identification in LC-MS-based proteomics, it is more difficult to identify metabolites on LC-MS platforms. At present, endogenous metabolite identification in untargeted metabolic analyses is mainly achieved through mass-based search followed by manual verifications. First, the m/z value of an ion is searched against database(s). Several databases have been assembled in recent years like Human Metabolome Database (HMDB) [15], Metlin [16], LipidMaps [17], and Madison Metabolomics Consortium Database (MMCD) [18] or more general chemical databases like PubChem or ChemSpider. Metabolites having masses within pre-specified tolerance range of the query mass are retrieved from these databases. These are putative identifications. However, the mass-based search can seldom provide unique identifications for the ions of interest.

To verify the mass-based search results, authentic compounds of those putative identifications are subjected to MS or tandem MS experiments together with the sample. By comparing the retention times or tandem MS spectra of the authentic compounds with the ions of interest in the sample, the identities of the metabolites can be confirmed.

## Methods

In analysis of LC-MS data from multiple experiments, the ions selected from each experiment are compared to determine overlapping ions. For example, the software tool metaXCMS [9] performs second-order analysis of untargeted metabolomic data from multiple sample groups representing different models of the same phenotype. The pairs of sample groups are first analyzed with XCMS and the output files are subsequently input into metaXCMS where they are realigned, statistically evaluated and compared for shared differences.

**Figure 1:**
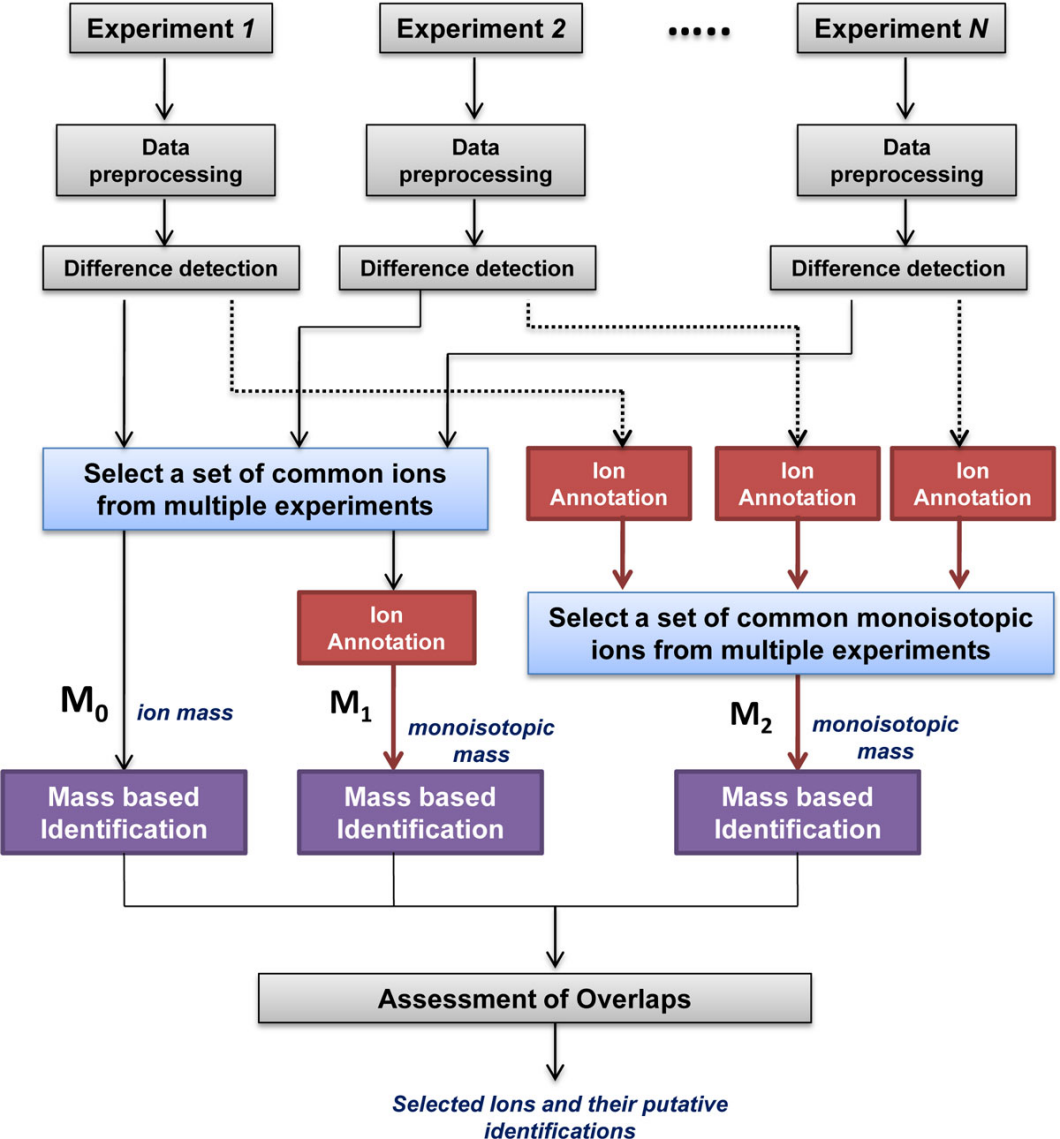
**Analysis of LC-MS data**. (M_0_) Traditional method. (M_1 _& M_2_) Ion annotation-assisted method.

The disadvantage of the traditional approach depicted in Figure 1 (M_0_) is the lack of recognition of isotopes, adducts, and fragments. For example, if these derivative ions are selected as significant, they will be searched against a database for identification as separate ions and will result in an inaccurate identification or no identification at all. Also, unless the same derivative ions are selected across experiments, their overlaps will be missed due to difference in their ion masses. However, with the help of ion annotation, such overlaps can be detected (see M_1 _and M_2 _in Figure 1). This ion annotation-assisted method is illustrated in the following sections.

### Ion annotation

Ion annotation is a procedure to recognize group of ions which are likely to originate from the same compound. In LC-MS based metabolomics, one metabolite is often represented by multiple peaks with distinct m/z values but at similar retention times. Recognition of those peaks from the same metabolite can facilitate the metabolite identification.

Generally, one metabolite can generate three types of ions in LC-MS data: adducts, isotopes, and in-source fragments. Adduct ion is "an ion formed by interaction of two species, usually an ion and a molecule, and often within the ion source, to form an ion containing all the constituent atoms of one species as well as an additional atom or atoms" [19]. The most common adduct ions in LC-MS are protonated ion [M+H]^+ ^or deprotonated ion [M-H]^- ^(although deprotonated ion is the loss of a proton rather than addition, it is generally considered as adduct). In addition, there could be other types of adducts, such as sodium adduct, potassium adduct, etc. Some of the most common forms of adducts are listed in Table 1 while more complete information concerning adduct in mass spectrometry can be found in [20,21].

**Table 1:** Common types of adducts in LC-MS

Ionization	Formation	Ion Mass
	[M+H]^+^	m+1.0073
	[M+2H]^2+^	m/2+1.0073
Positive	[M+Na]^+^	m+22.9892
	[M+K]^+^	m+38.9632
	[M+NH4]^+^	m+18.03382

	[M-H]^-^	m-1.0073
Negative	[M-2H]^2-^	m/2-1.0073
	[M-2H+Na]^-^	m+20.9747
	[M-2H+K]^-^	m+36.9486

M is the molecule with molecular weight m

Isotopes are variants of atoms of the same chemical elements, which have the same number of protons but different number of neutrons. As a result, the atoms of the same element may have different masses depending on the number of neutrons they have. Common metabolites are composed of elements carbon (C), hydrogen (H), nitrogen (N), oxygen (O), phosphor (P), and sulfur (S). Most of them have at least one naturally-existing, stable isotope. So metabolites are usually a mixture of several isotopic species. During mass spectrometry analysis, different isotopic species are separated, which will generate a series of peaks separated on m/z by around one Da difference. Among them, the peak with the lowest m/z is defined as the monoisotopic peak.

The third type of ions is caused by in-source fragments. Although ESI is generally considered as a soft-ionization approach which mainly generates intact molecular ion. Fragmentation may still happen during ionization. One common in-source fragments is water-loss fragment [M+H-H2O]^+ ^or [M-H-H2O]^-^, where a water molecule is lost during the ionization process.

Different adducts/isotopes/water-loss products of the same compound theoretically share the same retention time in chromatograms. As long as the scan rate is properly adjusted and enough scanning points are acquired to define the chromatographic peaks, the ions from the same compound share similar-shaped elution profiles which can be represented by their extracted ion chromatograms (EICs). Thus ion annotation can be accomplished by clustering similar elution profiles together. Different ion formations of the same metabolite will differ in their m/z values. The observed m/z (*X*) of an ion derived from a metabolite with a monoisotopic molecular weight M can be calculated as

$$x=\frac{nM+\alpha +\beta M_{neutron}}{z}$$

where *n *is the number of molecules in the ion, *α *is the mass of the adducts (or fragments), *M_neutron _*is the mass of the neutron, *β *is the extra number of neutrons in isotopes, and *z *is the charge of the ion. In LC-MS, many types of adducts and fragments are known. These include [M+H]^+^, [M+Na]^+^, [M+K]^+^, and [M+H-H2O]^+^. As a result, the m/z relationships between these known ion formations are often known a-priori.

An R-package CAMERA (Collection of Algorithms for MEtabolite pRofile Annotation) performs ion-annotation in two steps [22]. In the first step, the detected peaks with similar retention times are roughly grouped together using a sliding retention time window. Within each group, the EICs of the peaks are extracted and the peaks are clustered into smaller groups based on the Pearson correlation between their EICs. The m/z difference between each peak pair within a group is calculated and compared to known m/z relationships between different ion formations. The two ions are considered to come from the same compound if their m/z difference can be explained by one of the known m/z relationships.

Table 2 represents an example of ion masses grouped together using CAMERA. The ions in the table are represented by the same monoisotopic mass. We can see isotopes as well as adducts being grouped under the same monoisotopic mass. For example, ion D is the monoisotopic ion that represents glycochenodeoxycholic acid (GCDCA). Ion E is an isotope of ion D. Ion A represents neutral loss. Ions B and C are isotopes of ion A.

**Table 2:** Ion masses grouped together under monoisotopic mass 450

Ions	M/Z	RT	Isotopes	Adducts	Monoisotopic mass
A	432.311	227.13	[M]+	[M+H-H20]+ 449.316	450.3219
B	433.314	227.13	[M+1]+		450.3219
C	434.318	227.13	[M+2]+		450.3219
D	450.322	227.29	[M]+	[M+H]+ 449.316	450.3219
E	451.325	227.16	[M+1]+		450.3219
F	472.304	227.44		[M+Na]+ 449.316	450.3219

### Ion annotation-assisted method

The ion annotation information can be used to reduce the ion list by clustering the isotopic ions, adducts, and fragments represented by the same metabolite into groups by its monoisotopic mass (Figure 1). This will facilitate the comparison of ions selected across multiple experiments by allowing us to compare their monoisotopic mass instead of their individual masses. Specifically, the isotopes, fragments or adducts which belong to the same group will be clustered and given the same monoisotopic mass. This monoisotopic mass will then be used to compare the ions from multiple experiments and for subsequent mass-based metabolite identification. We explored the idea of combining the common ions after difference detection and performing ion annotation just prior to identification. The will help in selecting ions with the same mass and retention time that may have been missed, if annotated as different monoisotopes when ion annotation is performed separately for each experiment. A combination of these two methods will be an inclusive list of ions where ion annotation is performed prior or after comparing ions from multiple experiments.

After grouping ions together by ion annotation, the exact monoisotopic masses of these compounds can be calculated. The calculated masses will be a reduced list, which can be used to search against metabolite databases. An in-house software tool is developed to allow simultaneous search against four major metabolite databases: HMDB, Metlin, MMCD and LipidMaps. The same metabolite may appear in more than one database, the results from different databases are merged together based on the InChI Key of the retrieved metabolites. The InChI Key is the hashed version of International Chemical Identifier (InChI) and contains information about molecular formula, atom connection, and stereochemistry information of a compound. The merged results are used as the putative identifications for the ions of interest.

### LC-MS data from case-control studies

In this paper, we demonstrate the application of our proposed ion annotation-assisted method through two LC-MS datasets (Dataset 1 and Dataset 2) from our metabolomics biomarker discovery studies performed using Waters UPLC-QToF Premier instrument on human serum samples representing two distinct biological groups (cases and controls). A separate manuscript detailing the studies is in preparation.

Dataset 1 and Dataset 2 represent serum samples from cases and controls collected at different geographic locations. The two datasets were generated following the same sample preparation method and by using the Waters UPLC-QToF Premier instrument for each experiment. This gives us the opportunity to compare the ions selected from different studies for the same disease group.

#### Dataset 1

This dataset was generated from sera of 78 cases and 184 controls in three separate experiments (Exp. 1, Exp. 2, and Exp. 3) spanning across months, with different number of samples from cases and controls in each experiment. Both positive and negative ionization modes were used. Exp. 1 consisted of 60 cases and 129 controls, Exp. 2 had 13 cases and 50 controls, and Exp. 3 consisted of 5 cases and 5 controls.

#### Dataset 2

This dataset was generated from sera of 40 cases and 50 controls in four experiments (Exp. 1, Exp. 2, Exp. 3, and Exp. 4) run consecutively over a period of several days. Exp. 1 consisted of 20 cases and 25 controls. Exp. 2 consisted of the same samples from Exp. 1, processed in the reverse order. Exp. 3 consisted of separate 20 cases and 25 controls and Exp. 4 consisted of the same samples as in Exp. 3, but processed in the reverse order. The data was generated for both positive and negative ionization modes.

## Results and discussion

This section describes the results obtained by analyzing Datasets 1 and 2 using the traditional method and our proposed ion annotation-assisted method.

### LC-MS data preprocessing and difference detection

The raw data obtained from the UPLC-QToF machine were converted into Network Common Data Form (NetCDF) format using the MassLynx software (Waters Corp, Milford, MA). We used the XCMS package [4] to preprocess the three LC-MS datasets separately. To enable further analysis and visualization of data, all *m/z *values were binned to fixed *m/z *values with a bin size of 100 ppm. As a result, the data were transformed into a two-dimensional matrix of ions with specific *RT *and *m/z *values and columns represent the samples. A list of all ions in each sample was compiled. After detecting ions in individual samples, they were aligned across samples in each experiment to allow calculation of retention time deviations and relative ion intensity comparison.

For difference detection, in-house developed MATLAB (Natick, MA) and R scripts were used on the basis of parametric and non-parametric statistical tests (t-test and the Wilcoxon rank-sum test). In both statistical tests, we calculated the false discovery rate (FDR) to correct for multiple testing. Ions with q-value < 0.1 by either t-test or the Wilcoxon rank-sum test were selected.

### Ion annotation-assisted analysis of LC-MS data from multiple experiments

We applied three methods (M_0_, M_1_, and M_2_) depicted in Figure 1, to compare the ion masses across the multiple experiments. In M_0_, after difference detection, a set of common ions was selected based on ion mass and retention time. This selected list was then used directly for mass-based identification. M_1 _uses the ion mass list obtained from M_0 _and performs ion annotation on this list to cluster the ions into a list of monoisotopic masses. In M_2_, ion annotation is first performed on the list of ion masses obtained after difference detection from each experiment. This will result in a list of monoisotopic masses which are significant in each experiment. These monoisotopic masses are then used to select a set of common ions which are present in at least two of the three experiments. The retention time and the sign of the fold change are also taken into consideration when comparing. We used a tolerance of 15 seconds for retention time and a tolerance of 10 ppm for m/z. We required ions to be either up or down regulated in the experiments to be selected. The putative identification lists obtained from M_0_, M_1_, and M_2 _are then assessed for overlaps and differences among them. Through manual assessment, we selected a list of ions and their putative identifications for further verification of the identities of the metabolites.

#### Dataset 1

In Exp. 1, Exp. 2, and Exp. 3, from Dataset 1, we detected 1587, 3231, and 613 ions in the positive mode, respectively. In the negative mode, 942, 1210, and 392 ions were detected in Exp. 1, Exp. 2, and Exp. 3, respectively. From each experiment, we selected ions with significant difference between cases and controls. The ion lists from the three experiments were compared to determine overlapping ions using the three methods (M_o_, M_1_, and M_2_) depicted in Figure 1. Ions detected in each experiment and those selected by statistical methods are presented in Table 3. Table 4 presents ions overlapping in at least two of the three experiments.

**Table 3:** Number of ions detected in each of the three experiments and those selected by statistical analysis in Dataset 1

Mode	**Exp**.	# of ions detected	# of ions selected by Wilcoxon rank-sum test (q-value < 0.1)	# of ions selected by t-test (q-value < 0.1)	# of ions selected (Wilcoxon rank-sum test or t-test)
	Exp. 1	1587	187	171	226
Positive	Exp. 2	3231	82	0	82
	Exp. 3	613	133	122	168

	Exp. 1	942	676	622	714
Negative	Exp. 2	1210	23	0	23
	Exp. 3	392	0	48	48

**Table 4:** The number of ions overlapping between experiments in Dataset 1

	Overlapping ion masses		Overlapping monoisotopic ion masses					
	M_0_		M_1_		M_2_		Selected Ions	
Mode	2/3	3/3	2/3	3/3	2/3	3/3	2/3	3/3
Positive	46 (27)	3 (2)	23 (15)	1 (1)	16 (12)	2 (2)	23 (15)	2 (2)
Negative	16 (10)	0	12 (8)	0	13 (9)	0	13 (9)	0
Total	59 (34)	3 (2)	32 (20)	1 (1)	26 (18)	2 (2)	33 (21)	2 (2)

M_1 _and M_2 _use only monoisotopic ion masses; hence cannot be compared with M_0 _using individual ion masses. The number of ions overlapping in at least 2 out of 3 (2/3) and all three (3/3) experiments using M_0_, M_1_, and M_2 _are shown. The numbers in parentheses show the corresponding putative identifications.

Considering ions overlapping in at least two out of three experiments, we found 46 ion masses in positive mode and 16 ion masses in negative mode using M_0_. Three ion masses were found to be overlapping in all three experiments. The in-house software tool was then used for mass-based identification. From the total (positive and negative mode combined) ion list selected by M_0_, we found putative metabolite identifications for 34 out of 59 ion masses. There were three ion masses whose putative identifications were repeated, indicating that these metabolites were selected in both modes. It should be noted that in M_0_, isotopes or adducts which belong to the same monoisotope are treated as separate ion masses and can have different putative metabolite identifications or no identifications. This can lead to misinterpretation of the metabolites selected using M_0_.

Comparing the results from M_0 _with those from M_1_, we observed that the 59 individual ion masses selected by M_0 _represent only 32 monoisotopic masses of which 20 have putative identifications. Thus, the putative identifications previously found by M_0 _for 34 ions actually represent only 20 metabolites. We observed that the remaining 14 ions were assigned wrong putative identifications, because they are isotopes, adducts, or fragments. On the other hand, M_1 _and M_2 _were able to eliminate such wrong identifications, because it uses ion annotation that clusters together ions into their monoisotopic masses.

Considering ions overlapping in all three experiments, M_0 _found three ion masses, of which two had putative identifications. Through M_1_, we found that these three ions represent only one monoisotopic mass. M_2 _selected an extra monoisotopic mass that was missed by other two methods and this ion was selected in all three experiments. Table 4 presents the number of overlapping ions (positive and negative modes) and monoisotopic masses as well as the number of ions with putative identifications found by M_0_, M_1_, and M_2 _in at least two out of the three experiments and all three experiments.

#### Dataset 2

This dataset consisted of four experiments, Exp.1, Exp.2, Exp.3, and Exp.4. In Exp. 1, Exp. 2, Exp. 3, and Exp. 4, we detected 724, 790, 864, and 826 ions in the positive mode, respectively. Similarly, in the negative mode, 534, 487, 564, and 505 ions were detected in Exp. 1, Exp. 2, Exp. 3 and Exp. 4, respectively. Four pair-wise comparisons were performed to select a set of common ions among pair of two experiments involving independent samples. The pair-wise comparisons were: (i) Exp.1 vs. Exp.3, (ii) Exp.1 vs. Exp.4, (iii) Exp.2 vs. Exp. 3, and (iv) Exp.2 vs. Exp.4. The pair-wise comparisons, Exp.1 vs. Exp.2 and Exp.3 vs. Exp.4 were not considered as the samples in those pairs are the same. In each pair-wise comparison, we retained only those ions that are present in both experiments. Other ions were excluded during difference detection by statistical methods. Difference detection was applied to each pair to select a set of statistically significant ions. Ions with q-value < 0.1 by either t-test or the Wilcoxon rank-sum test were selected. Table 5 shows the number of ions detected as well as those selected by statistical methods in each experiment. Table 6 presents the ions selected commonly by a pair of experiments involving two independent set of samples. Specifically, an ion is selected for mass based identification if it is statistically significant in one of the four pairs of experiments (i.e., Exp.1 vs. Exp.3, Exp.1 vs. Exp.4, Exp.2 vs. Exp. 3, or Exp.2 vs. Exp.4).

**Table 5:** Number of ions detected in each of the four experiments and those selected by statistical analysis in Dataset 2

Mode	**Exp**.	# of ions detected	# of ions selected by Wilcoxon rank- sum test (q-value < 0.1)	# of ions selected by t-test (q-value < 0.1)	# of ions selected (t-test or Wilcoxon rank-sum test)
	Exp. 1	724	7	4	7
Positive	Exp. 2	790	46	52	64
	Exp. 3	864	201	99	201
	Exp. 4	826	0	1	1

	Exp. 1	534	4	4	4
Negative	Exp. 2	487	5	5	5
	Exp. 3	564	71	54	83
	Exp. 4	505	2	5	5

**Table 6:** The number of ions overlapping between two independent sample sets among four experiments in Dataset 2

	Overlapping ion masses	Overlapping monoisotopic ion masses		
Mode	M_0_	M_1_	M_2_	Selected Ions
Positive	24 (10)	14 (7)	15 (8)	16 (8)
Negative	1 (1)	1 (1)	1 (1)	1 (1)
Total	24 (10)	15 (8)	16 (9)	17 (9)

M_1 _and M_2 _use only monoisotopic ion masses, hence cannot be compared with M_0 _using individual ion masses. The selected ions are statistically significant in Exp.1 vs. Exp.3, Exp.1 vs. Exp.4, Exp.2 vs. Exp. 3, or Exp.2 vs. Exp.4 pair-wise comparisons in Dataset 2. The numbers in parentheses show the corresponding putative identifications.

Comparing the results from M_0 _with those from M_1 _on Dataset 2, we observed that the 25 individual ion masses selected by M_0 _represent only 15 monoisotopic masses, of which 8 have putative identifications. Thus, the putative identifications previously found by M_0 _for 10 ions actually represent only 8 metabolites. The remaining 2 ions were assigned wrong putative identifications, because they are isotopes, adducts, or fragments. M_1 _and M_2 _were able to eliminate such wrong identifications. M_2 _captured two additional overlapping monoisotopic ion masses that were missed by M_0 _and M_1_. There was an additional ion selected by M_1_, which was missed by M_2_. This ion was statistically significant in the pair-wise comparison of Exp.2 vs. Exp.3, but was wrongly annotated in one of the experiment. M_1 _was able to capture this ion since the ion annotation was performed after the selection of overlapping ions. This is a limitation of M_2_, which is impacted by wrongly annotated ions. Our future work focuses on reducing this limitation. To include such missed ions due to wrong ion annotation, we use manual assessment of the results from all three methods to create a list of selected ions with their corresponding putative identifications for further verification. Table 6 presents the number of overlapping ions (positive and negative modes) and monoisotopic masses as well as the number of ions with putative identifications found by M_0_, M_1_, and M_2 _in Dataset 2.

### Comparison of Dataset 1 and Dataset 2

Dataset 1 and Dataset 2 consisted of serum samples of cases and controls from the same disease, collected at different laboratories. From Dataset 1, we selected 33 ions of which 21 ions have putative identifications. From Dataset 2, we chose 17 ions with 9 ions having putative identifications. We found two ions with putative identifications overlapping between the two datasets. These metabolites are good candidates for further biomarker validation.

## Conclusion

Analysis of mass spectrometric data continues to be an important area due to the large amount of data being generated in various metabolomic studies addressing similar or related hypotheses. Thus, computational tools are needed for comparison or integration of multiple experiments.

The ion annotation-assisted analysis of LC-MS based metabolomic experiments yields useful information about the detected ion masses. For example, in this study we observe that the number of putative identifications obtained without the use of ion annotation is reduced significantly following ion annotations. This is primarily due to different putative identifications assigned to multiple ions despite sharing the same monoisotopic mass. Thus, ion annotation-assisted analysis helps to reduce the required manual curation effort as well as the subsequent analysis to verify the identity of the metabolites. The use of ion annotation helps to increase the reliability of overlapping monoisotopic ions that have putative identifications. It also helps to select those metabolites that may have been missed by the traditional method. For example, we were able to identify an extra metabolite using M_2 _in Dataset 1 and two extra metabolites in Dataset 2. These metabolites were missed by M_0 _and M_1_. Thus, in studies involving multiple experiments, the proposed ion annotation-assisted method will be useful to identify metabolites which overlap across multiple experiments with more coverage and greater reliability. Verification of the identity of the ions with putative identifications is underway. This verification and subsequent quantitation by a targeted analysis are necessary to validate the improvements achieved by our analysis method.

Our future goal is to investigate how the ion annotation can be performed on multiple experiments together rather than treating each experiment separately. This will ensure that ions represented by same mass and retention time to share the same monoisotopic mass in multiple experiments. Also, we plan to develop a method that automatically integrates the results from M_0_, M_1_, and M_2 _to take advantage of the benefits of each method.

## Competing interests

The authors declare that they have no competing interests.

## Authors' contributions

RSV drafted the manuscript and participated in the statistical analysis. BZ performed the preprocessing and ion annotation using CAMERA. MRNR and YZ participated in the statistical analysis. HWR guided the study and drafted the manuscript. All authors read and approved the final manuscript.

## References

[B1] Chen C Gonzalez FJ Idle JR LC-MS-Based Metabolomics in Drug Metabolism Drug Metabolism Reviews 2007 39 581 597 10.1080/03602530701497804 17786640 PMC2140249

[B2] Jonsson P Gullberg J Nordström A Kusano M Kowalczyk M Sjöström M Moritz T A Strategy for Identifying Differences in Large Series of Metabolomic Samples Analyzed by GC/MS Analytical Chemistry 2004 76 1738 1745 10.1021/ac0352427 15018577

[B3] Tikunov Y Lommen A de Vos CH Verhoeven HA Bino RJ Hall RD Bovy AG A novel approach for nontargeted data analysis for metabolomics. Large-scale profiling of tomato fruit volatiles Plant Physiol 2005 139 1125 1137 10.1104/pp.105.068130 16286451 PMC1283752

[B4] Smith CA Want EJ O'Maille G Abagyan R Siuzdak G XCMS: processing mass spectrometry data for metabolite profiling using nonlinear peak alignment, matching, and identification Anal Chem 2006 78 779 787 10.1021/ac051437y 16448051

[B5] Xia J Psychogios N Young N Wishart DS MetaboAnalyst: a web server for metabolomic data analysis and interpretation Nucleic Acids Res 2009 37 W652 660 10.1093/nar/gkp356 19429898 PMC2703878

[B6] Pluskal T Castillo S Villar-Briones A Oresic M MZmine 2: modular framework for processing, visualizing, and analyzing mass spectrometry-based molecular profile data BMC Bioinformatics 2010 11 395 10.1186/1471-2105-11-395 20650010 PMC2918584

[B7] Katajamaa M Oresic M Processing methods for differential analysis of LC/MS profile data BMC Bioinformatics 2005 6 179 10.1186/1471-2105-6-179 16026613 PMC1187873

[B8] Katajamaa M Oresic M Data processing for mass spectrometry-based metabolomics J Chromatogr A 2007 1158 318 328 10.1016/j.chroma.2007.04.021 17466315

[B9] Scheltema R Decuypere S Dujardin J Watson D Jansen R Breitling R Simple data-reduction method for high-resolution LC-MS data in metabolomics Bioanalysis 2009 1 1551 1557 10.4155/bio.09.146 21083103

[B10] Tautenhahn R Böttcher C Neumann S Hochreiter S, Wagner R Annotation of LC/ESI-MS Mass Signals Bioinformatics Research and Development 2007 4414 Springer Berlin/Heidelberg 371 380 Lecture Notes in Computer Science 10.1007/978-3-540-71233-6_29

[B11] Bocker S Letzel MC Liptak Z Pervukhin A SIRIUS: decomposing isotope patterns for metabolite identification Bioinformatics 2009 25 218 224 10.1093/bioinformatics/btn603 19015140 PMC2639009

[B12] Jaitly N Mayampurath A Littlefield K Adkins JN Anderson GA Smith RD Decon2LS: An open-source software package for automated processing and visualization of high resolution mass spectrometry data BMC Bioinformatics 2009 10 87 10.1186/1471-2105-10-87 19292916 PMC2666663

[B13] Horn DM Zubarev RA McLafferty FW Automated reduction and interpretation of high resolution electrospray mass spectra of large molecules J Am Soc Mass Spectrom 2000 11 320 332 10.1016/S1044-0305(99)00157-9 10757168

[B14] Storey J Lovric M False discovery rates International Encyclopedia of Statistical Science 2011 1 Springer 1673

[B15] Wishart DS Knox C Guo AC Eisner R Young N Gautam B Hau DD Psychogios N Dong E Bouatra S HMDB: a knowledgebase for the human metabolome Nucl Acids Res 2009 37 D603 610 10.1093/nar/gkn810 18953024 PMC2686599

[B16] Smith CA Maille GO Want EJ Qin C Trauger SA Brandon TR Custodio DE Abagyan R Siuzdak G METLIN: A Metabolite Mass Spectral Database Therapeutic Drug Monitoring 2005 27 747 751 10.1097/01.ftd.0000179845.53213.39 16404815

[B17] Fahy E Sud M Cotter D Subramaniam S LIPID MAPS online tools for lipid research Nucleic Acids Res 2007 35 W606 612 10.1093/nar/gkm324 17584797 PMC1933166

[B18] Cui Q Lewis IA Hegeman AD Anderson ME Li J Schulte CF Westler WM Eghbalnia HR Sussman MR Markley JL Metabolite identification via the Madison Metabolomics Consortium Database Nat Biotechnol 2008 26 162 164 10.1038/nbt0208-162 18259166

[B19] McNaught AD Wilkinson A IUPAC:Compendium of Chemical Terminology 1997 2 Oxford:Blackwell Science

[B20] Huang N Siegel M Kruppa G Laukien F Automation of a Fourier transform ion cyclotron resonance mass spectrometer for acquisition, analysis, and e-mailing of high-resolution exact-mass electrospray ionization mass spectral data Journal of the American Society for Mass Spectrometry 1999 10 1166 1173 10.1016/S1044-0305(99)00089-6

[B21] Keller BO Sui J Young AB Whittal RM Interferences and contaminants encountered in modern mass spectrometry Analytica Chimica Acta 2008 627 71 81 10.1016/j.aca.2008.04.043 18790129

[B22] Tautenhahn R Böttcher C Neumann S Hochreiter S, Wagner R Annotation of LC/ESI-MS Mass Signals in Bioinformatics Research and Development 2007 4414 Springer Berlin/Heidelberg 371 380

